# The Effects of Poly-γ-Glutamic Acid on the Postharvest Physiology and Quality of Strawberry cv. Hongyan during Cold Storage

**DOI:** 10.3390/foods12152944

**Published:** 2023-08-03

**Authors:** Changjuan Shan, Yi Luo, Chen Yang, Xinxia Gao

**Affiliations:** School of Life Sciences, Henan Institute of Science and Technology, Xinxiang 453003, China; luoyi@stu.hist.edu.cn (Y.L.); zhangxq@stu.hist.edu.cn (C.Y.); gao@stu.hist.edu.cn (X.G.)

**Keywords:** antioxidant capacity, cell-wall-degrading enzyme, postharvest preservation, nutritional quality, strawberry

## Abstract

This study investigated the effects of poly-γ-glutamic acid (γ-PGA) on the postharvest physiology and quality of the strawberry cv. Hongyan during cold storage. The results showed that all concentrations of γ-PGA improved decay control and strawberry preservation by enhancing antioxidant capacity, delaying the softening process, and maintaining fruit quality, especially for 100 mg·L^−1^ γ-PGA. After 14 days of treatment, compared with control, 100 mg·L^−1^ γ-PGA decreased weight loss, decay rate, and index by 21.9%, 75.0%, and 66.7% and increased the total antioxidant capacity by 43.5% through antioxidant enzymes. In addition, 100 mg·L^−1^ γ-PGA increased fruit firmness by 53.6% by decreasing the activities of polygalacturonase, pectin lyase, cellulase, and β-galactosidase. In terms of color quality, 100 mg·L^−1^ γ-PGA improved the values of lightness and yellowness by 30.9% and 52.8%. As regards nutritional quality, 100 mg·L^−1^ γ-PGA increased the contents of protein, soluble sugars, vitamin C, and total phenols by 106.6%, 80.6%, 51.2%, and 78.4%. In terms of sensory quality, 100 mg·L^−1^ γ-PGA increased the soluble solids’ content by 19.0% and decreased the titrated acids’ content by 21.1%, which increased the sugar–acid ratio by 50.9%. Our findings suggest that 100 mg·L^−1^ γ-PGA can be used to improve the decay control and preservation of strawberry cv. Hongyan under cold storage.

## 1. Introduction

Strawberry fruit is rich in nutrients and phytochemicals, including proteins, sugars, vitamins, anthocyanins, and phenolics [1,2]. However, strawberries are very perishable. Thus, they often require special treatment after harvest to control decay and quality. It has been documented that many exogenous chemicals could significantly improve decay control and quality preservation, including chitosan, proline, putrescine, citrus essential oils, ozone, phytosulfokine, CO_2_, etc. [3,4,5,6,7,8,9]. Wang et al. [3] reported that a chitosan coating inhibited the softening process of strawberries under cold storage. Panou et al. [6] showed that ozone treatment maintained the quality and improved the shelf life of strawberries under cold storage. The above studies clearly indicate that we can maintain strawberry quality under cold storage by using exogenous chemicals.

Poly-γ-glutamic acid (γ-PGA) is a vital biopolymer produced through microbial fermentation. This kind of biopolymer is non-toxic, edible, biodegradable, and eco-friendly. It also shows advantages in film formation and fertilizer retention. Due to the above properties, γ-PGA has been used in many industrial fields, including food, biomedicine, and agriculture [10,11]. In agriculture, Xu et al. [12] found that γ-PGA improved wheat productivity and nitrogen-use efficiency. Liang et al. [13] reported that γ-PGA promoted soil’s water-holding capacity, water use efficiency, cotton growth, and yield. Bai et al. [14] found that γ-PGA enhanced the growth of Chinese cabbage by affecting the rhizosphere bacterial community. In addition, only a small number of studies reported γ-PGA application in food preservation. For example, γ-PGA hydrogel improved the storage life and quality of shiitake mushrooms [15]. γ-PGA showed a better cryoprotective effect in the frozen storage of grass carp surimi [16]. However, there is still no report on the application of γ-PGA during the cold storage of strawberries. Thus, it will be interesting to investigate the effects of γ-PGA on the postharvest physiology and quality of strawberries during cold storage, which can provide a theoretical basis for its application in strawberry preservation.

Strawberry preservation is closely related to antioxidant capacity, which can be indicated by the total antioxidant capacity and the activities of antioxidant enzymes [4,7,9,17,18]. Previous studies demonstrated that exogenous chemicals improved strawberry preservation by enhancing its antioxidant capacity. Bahmani et al. [4] reported that proline, chitosan, and proline-coated chitosan nanoparticles significantly increased the antioxidant capacity of strawberries under cold storage by enhancing superoxide dismutase (SOD) activity and decreasing the contents of hydrogen peroxide (H_2_O_2_) and malondialdehyde (MDA). Shehata et al. [7] showed that citrus essential oils improved the antioxidant capacity of strawberries during cold storage by enhancing catalase (CAT) activity. Regarding γ-PGA, Lei et al. [19] found that γ-PGA increased the antioxidant capacity of rape seedlings under cold stress by enhancing the activities of SOD, CAT, and peroxidase (POD). Xu et al. [20] reported that γ-PGA enhanced the antioxidant capacity of rape seedlings under drought stress by improving the activities of SOD, CAT, and ascorbate peroxidase (APX). The above studies indicate that the antioxidant capacity of strawberry fruits can be enhanced by using exogenous chemicals, including γ-PGA.

Quality maintenance is of great significance for strawberry preservation during cold storage. Previous studies showed that exogenous chemicals could maintain the quality of strawberries under cold storage [4,5,18,21,22]. Bal and Ürün [5] demonstrated that putrescine alone or in combination with chitosan maintained strawberry quality under cold storage, in terms of features such as weight loss; decay incidence; titratable acidity; and the contents of soluble solids (SS), vitamin C (Vc), total anthocyanins, and total phenolics. Duan et al. [23] reported that fucoidan and carboxymethyl fucoidan could maintain strawberry quality during cold storage, including decay incidence and Vc content. In addition, Wang et al. [3] found that a chitosan coating inhibited fruit softening in strawberries during cold storage by regulating the activities of enzymes responsible for cell wall degradation, which further reduced firmness loss. It has also been reported that γ-PGA hydrogel improved the quality of shiitake mushrooms [15]. The above studies suggest that we could reduce the quality loss of strawberries by using exogenous chemicals, including γ-PGA.

However, there is still no report on the effects of γ-PGA on the antioxidant capacity and quality of strawberries under cold storage. Thus, it will be very interesting to carry out this work. In the current study, we hypothesized that γ-PGA can enhance the antioxidant capacity and maintain the quality of strawberries under cold storage, which further improves strawberry preservation. To prove this hypothesis, we investigated the effects of γ-PGA on decay rates and indexes; weight loss; the activities of antioxidant enzymes and enzymes in the ascorbate–glutathione (AsA-GSH) cycle; the total antioxidant capacity; the contents of MDA and H_2_O_2_; firmness and the activities of the enzymes responsible for cell wall degradation; the color parameters L*, a*, and b*; nutritional quality indicators of soluble sugars; Vc; total phenolics; total anthocyanins and proteins; and the taste indicators SS, titratable acids (TAs), and the sugar–acid ratios (SARs) of strawberries during cold storage. Thus, the aim of the current study was to clarify the roles of γ-PGA in the decay control and quality preservation of strawberries under cold storage, which will provide new information for its application in the postharvest storage of strawberries.

## 2. Materials and Methods

### 2.1. Plant Material and Treatments

Strawberry *Fragaria* × *ananassa* Duch. cv. Hongyan was used for this experiment. Cultivar Hongyan has the advantages of being a large fruit, having a high yield, good taste, and suitable storage, which makes it an ideal variety for facility cultivation in China. Strawberry fruits with around 90% red areas were obtained from plants grown in the greenhouse (Xinxiang, Henan, China) and immediately transported to the laboratory after harvest. Fruits with similar maturities, sizes, and colors without visual defects and physical damage were selected for the whole experiment.

In order to select suitable doses and duration of γ-PGA treatment, we investigated the effects of different concentrations of γ-PGA (12.5, 25.0, 50, 100, and 200 mg·L^−1^) on the appearance and freshness of strawberries under cold storage for 14 days. Five groups of fruits were immersed in 12.5, 25.0, 50, 100, and 200 mg·L^−1^ γ-PGA for 1, 5, and 10 min and designated as γ-PGA-treated fruits. One group was only immersed in an equal volume of distilled water for 1, 5, and 10 min and designated as control. For each treatment, 24 fruits were randomly divided into 4 replicates, with 6 fruits per replicate. The abovementioned fruits were then air-dried at room temperature for 2 h. All fruits were packed in plastic boxes with holes in the lid (6 fruits with a total weight of about 150 g per box). The length, width, and height of plastic boxes were 17.5 cm, 15.5 cm, and 7.5 cm, respectively. Then, all fruits packed in plastic boxes were stored at 4 °C with 90% relative humidity for 14 days. The results showed that treatments with different concentrations of γ-PGA for 1 min had no evident effects on fruit appearance and freshness. Treatments with 12.5 and 25.0 mg·L^−1^ γ-PGA for 5 and 10 min also had no evident effects on fruit appearance and freshness, while the fruits treated with 50, 100, and 200 mg·L^−1^ γ-PGA for 5 and 10 min all showed better appearance and freshness during cold storage. In addition, there were no evident differences between the fruits treated with the same concentration of γ-PGA for 5 and 10 min. Thus, we chose 50, 100, and 200 mg·L^−1^ γ-PGA as the doses of treatment and 5 min as the duration of treatment.

Different concentrations of γ-PGA (50, 100, and 200 mg·L^−1^) were prepared by dissolving 50, 100, and 200 mg γ-PGA in 1 L of distilled water. Food-grade γ-PGA (99% purity) with a molecular weight of 700 thousand was purchased from Xi’an Lavia Biotechnology Co., Ltd. (Xi’an, China). The selected fruits were randomly divided into four groups. Three groups were immersed in 50, 100, and 200 mg·L^−1^ γ-PGA for 5 min and designated as γ-PGA-treated fruits. One group was only immersed in an equal volume of distilled water for 5 min and designated as control. Then, the abovementioned fruits were air-dried at room temperature for 2 h. All fruits were packed in plastic boxes (6 fruits per box) and stored at 4 °C with 90% relative humidity for 14 days. In order to analyze the physiological and biochemical indicators, sampling was performed at 0, 4, 8, and 14 days of treatment. At each sampling point, 24 fruits were randomly divided into 4 replicates for each treatment, with 6 fruits per replicate. Samples were frozen in liquid nitrogen and then kept at −80 °C. To determine the weight loss, decay rate, and index of the fruits, the 24 fruits were also randomly divided into 4 replicates for each treatment, with 6 fruits per replicate. To show the effects of γ-PGA on the appearance and freshness of strawberries during cold storage, photos of the different treatments were taken at 0, 4, 8, and 14 days of treatment.

### 2.2. Assays of Weight Loss, Decay Rate, and Index of Fruit

For the determination of weight loss, the initial weight of fruits under each treatment was recorded at 0 days of treatment. After 4, 8, and 14 days of treatment, the final weight of the same fruits under each treatment was recorded. The weight loss was calculated by using Equation (1).

Weight loss (%) = [(Initial weight − Final weight)/Initial weight] × 100(1)

After 4, 8, and 14 days of treatment, fruits with visible spoilage were all considered to be decayed. The decay rate was calculated by using Equation (2) [4]. The decay index was measured according to the fruit decay degree by using Equation (3) [24]. In this study, we divided the decay degree into 4 grades according to fruit decay area, namely grade 0: no decay, grade 1: decay area less than 10%; grade 2: decay area between 10% and 30%; and grade 3: decay area more than 30%. Each treatment was performed in all four replicates.

Decay rate (%) = (number of decayed fruits/total fruit number) × 100(2)

Decay index (%) = [Ʃ(grade × fruit number per grade)/(total fruit number × 3)] × 100(3)

### 2.3. Assays of Antioxidant Enzymes in Fruit

At 0, 4, 8, and 14 days of treatment, the activities of SOD (EC 1.15.1.1), POD (EC 1.11.1.7), and CAT (EC 1.11.1.6) were analyzed according to Giannopolitis and Ries [25], Scebba et al. [26] and Kato and Shimizu [27], respectively. The specific activities of the abovementioned enzymes were expressed as U g^−1^ fresh weight (FW). Each treatment was performed in all four replicates.

### 2.4. Assays of Enzymes in AsA-GSH Cycle in Fruit

At 0, 4, 8, and 14 days of treatment, the activities of ascorbate peroxidase (APX, EC 1.11.1.11), glutathione reductase (GR, EC 1.6.4.2), monodehydroascorbate reductase (MDHAR, EC 1.6.5.4), and dehydroascorbate reductase (DHAR, EC 1.8.5.1) were analyzed according to Shan and Liang [28]. The specific activities for the abovementioned enzymes were expressed as U g^−1^ FW. Each treatment was performed in all four replicates.

### 2.5. Assays of Total Antioxidant Capacity and the Contents of MDA and H_2_O_2_ in Fruit

At 0, 4, 8, and 14 days of treatment, the total antioxidant capacity was determined according to the operating instructions of the total antioxidant capacity test kit (Solarbio, Solarbio Technology Co., Ltd., Beijing, China). MDA content was measured according to Heath and Packer [29]. H_2_O_2_ content was measured according to Brennan and Frenkel [30] and calculated from the standardized curve of H_2_O_2_. Each treatment was performed in all four replicates.

### 2.6. Assay of Fruit Firmness

At 0, 4, 8, and 14 days of treatment, fruit firmness was measured by using a digital force gauge (FR-5120, QA Supplies, Norfolk, VA, USA) equipped with a 3 mm diameter cylindrical probe. Three different locations were used to measure the firmness around the equatorial area. Firmness was expressed as kg cm^−2^. Each treatment was performed in all four replicates.

### 2.7. Assays of Enzymes Responsible for Cell Wall Degradation in Fruit

At 0, 4, 8, and 14 days of treatment, the activities of enzymes responsible for cell wall degradation were measured. The extraction and activity determination of PG, PL, CL, and β-Gal were performed according to the operating instructions of the corresponding test kits (Solarbio, Solarbio Technology Co., Ltd., Beijing, China). Each treatment was performed in all four replicates.

### 2.8. Measurement of Fruit Color Parameters

At 0, 4, 8, and 14 days of treatment, the color parameters of the fruit surface were measured by using a hand-held colorimeter (CS-10, CHNspec, Hangzhou, China). Calibration was performed by using a white plate. For each treatment, values of L* (lightness), a* (redness), and b* (yellowness) were recorded. Each treatment was performed in all four replicates.

### 2.9. Determination of Fruit Nutritional Quality

At 0, 4, 8, and 14 days of treatment, soluble sugars were determined according to Wei [31]. The content of Vc was measured according to Hodges et al. [32]. Total phenol content was analyzed according to Swain and Hillis [33] and is presented as grams of gallic acid equivalents per kilogram of fruit (mg GAE g^−1^ FW). Total anthocyanin content was analyzed according to the operating instructions of the corresponding test kits (Solarbio, Solarbio Technology Co., Ltd., Beijing, China) and is presented as μmol g^−1^ FW. Protein content was analyzed according to Bradford [34]. Each treatment was performed in all four replicates.

### 2.10. Determination of Fruit Sugar−Acid Ratio

At 0, 4, 8, and 14 days of treatment, the content of soluble solids (SSs) was determined using a hand-held saccharimeter (PAL-1, Atago, Tokyo, Japan). Titratable acids (TAs) were measured according to Li [35]. The sugar−acid ratio (SAR) was expressed as the ratio of SS content to TA content [36]. Each treatment was performed in all four replicates.

### 2.11. Statistical Analysis

The results are the mean of the four replicates. Statistical analyses were performed using a one-way analysis of variance with IBM SPSS Statistics software 26.0 (SPSS, Inc., Chicago, IL, USA). Differences between treatments were compared using Duncan’s multiple range test at a 5% level of significance.

## 3. Results

### 3.1. Effects of γ-PGA on the Appearance and Freshness of Strawberry under Cold Storage

**Figure 1:**
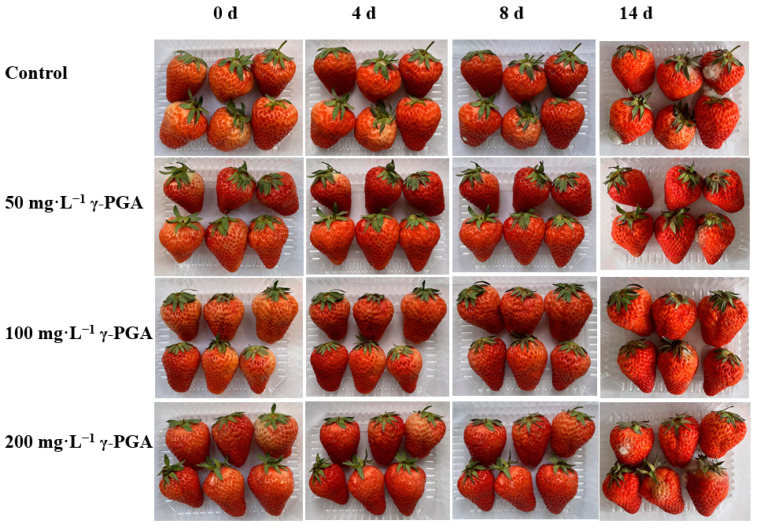
Effect of γ-PGA on the appearance of strawberries under cold storage. Fruits were treated with distilled water (control), 50 mg·L^−1^ γ-PGA, 100 mg·L^−1^ γ-PGA, and 200 mg·L^−1^ γ-PGA. Then, all fruits were stored at 4 °C for 14 days. Different lowercased letters represent significant differences among different treatments at *p* < 0.05.

**Figure 2:**
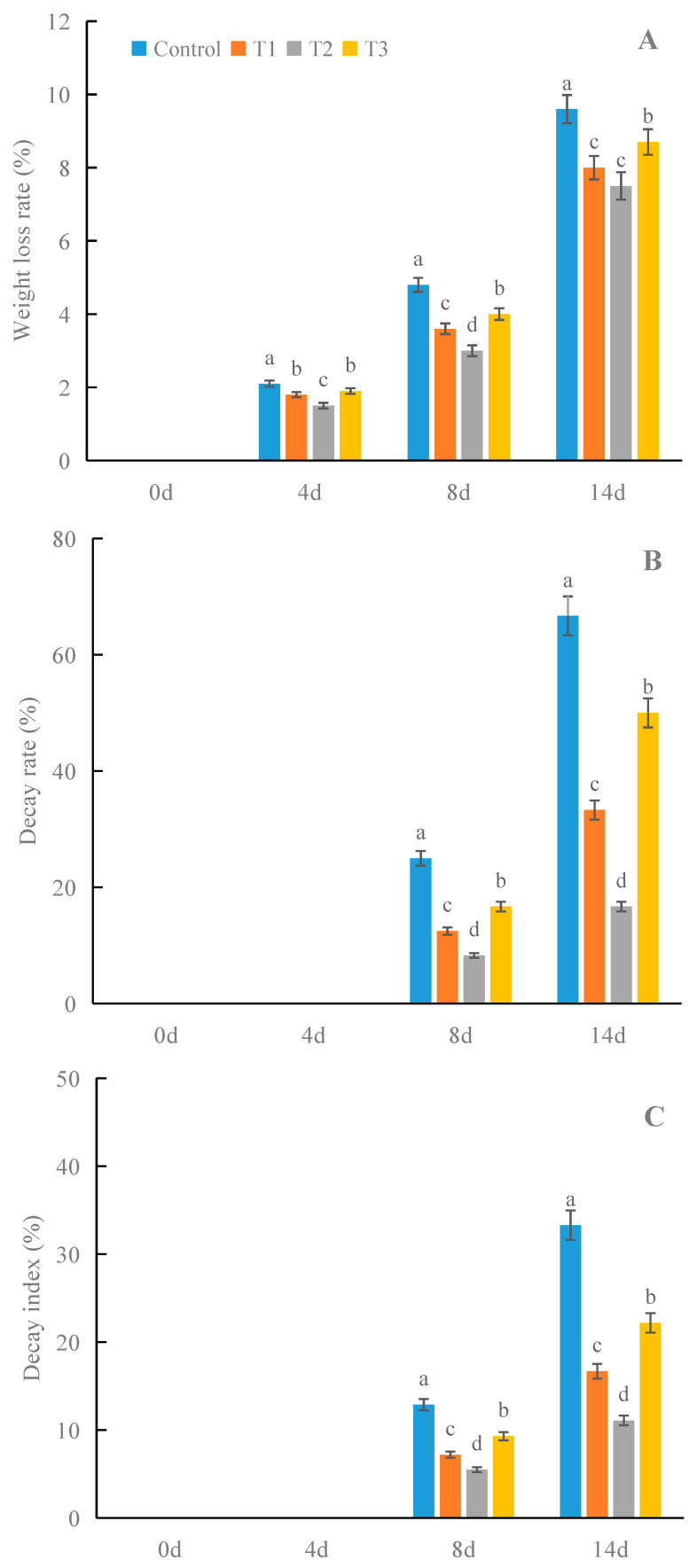
Effects of γ-PGA on the weight loss (**A**), decay rate (**B**), and index (**C**) of strawberries under cold storage. The fruits were treated as follows: control—distilled water; T1—50 mg·L^−1^ γ-PGA; T2—100 mg·L^−1^ γ-PGA; and T3—200 mg·L^−1^ γ-PGA. All fruits were then stored at 4 °C for 14 days. Different lowercased letters represent significant differences among different treatments at *p* < 0.05.

Compared with the control, all concentrations of γ-PGA showed better effects on the appearance and maintaining the freshness of strawberries during cold storage (Figure 1). After 8 days of treatment, the control group began to show decay and visible infection phenomenon. Compared with the control, the fruits treated with different concentrations of γ-PGA only showed slight decay. In particular, in the group treated with 100 mg·L^−1^ γ-PGA, only one fruit showed slight decay. After 14 days of treatment, the control showed serious decay and visible infection phenomenon. The decay and infection of the groups subjected to the different γ-PGA treatments were lower than that of the control. In the group treated with 100 mg·L^−1^ γ-PGA treatment, only one fruit showed decay and visible infection phenomenon. In addition, γ-PGA significantly decreased the weight loss and decay rate and index of fruits during cold storage, especially for 100 mg·L^−1^ γ-PGA (Figure 2). Compared with the control, 100 mg·L^−1^ γ-PGA decreased the weight loss and decay rate and index by 37.5%, 66.8%, and 57.4%, respectively, after 8 days of treatment. After 14 days of treatment, 100 mg·L^−1^ γ-PGA decreased the weight loss and decay rate and index by 21.9%, 75.0%, and 66.7%, respectively. The above results indicated that γ-PGA improved the appearance and freshness of strawberries under cold storage.

### 3.2. Effects of γ-PGA on the Activities of SOD, POD, and CAT in Fruit under Cold Storage

**Figure 3:**
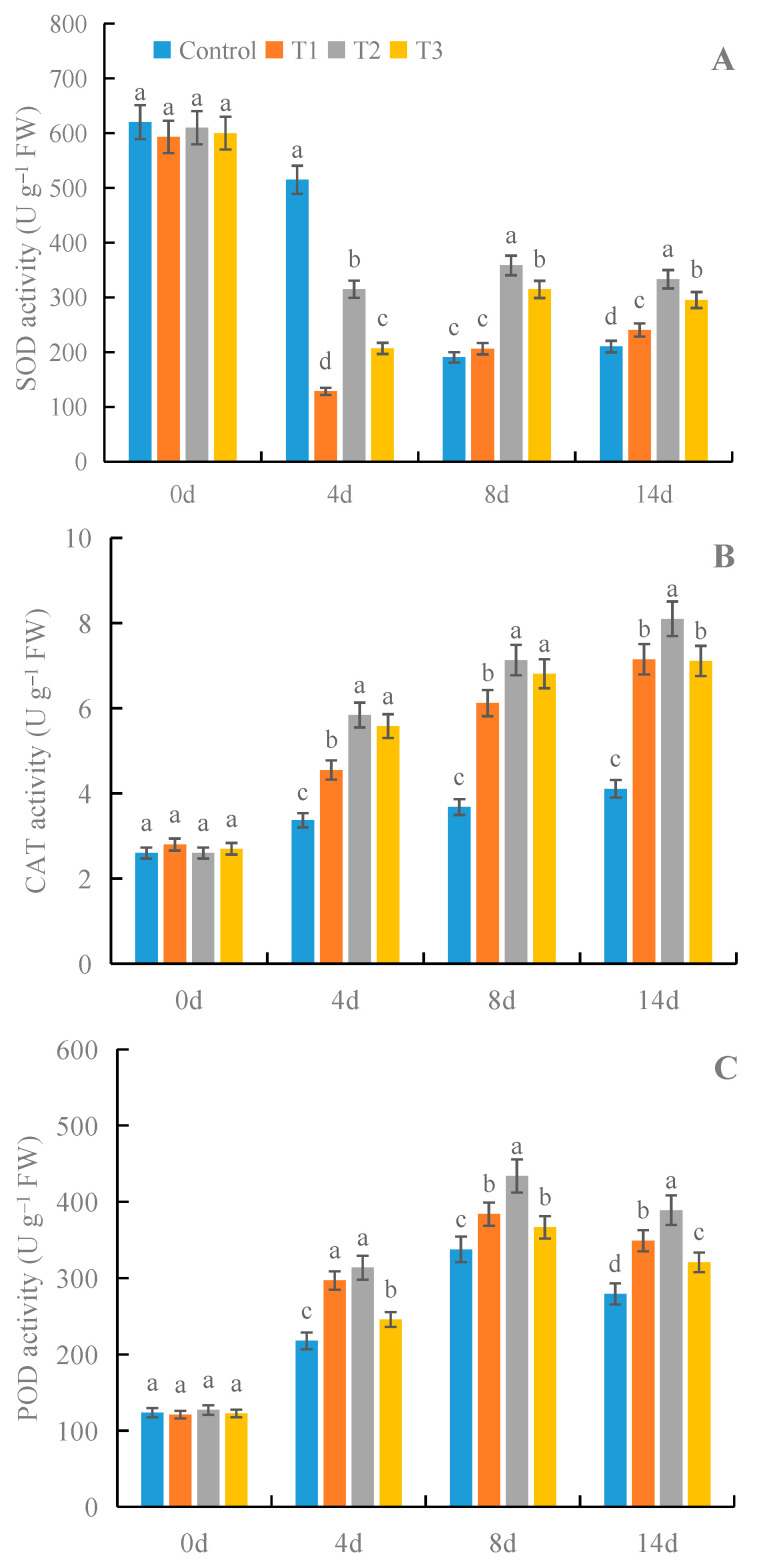
Effects of γ-PGA on SOD (**A**), CAT (**B**), and POD (**C**) activities in strawberries under cold storage. The fruits were treated as follows: control—distilled water; T1—50 mg·L^−1^ γ-PGA; T2—100 mg·L^−1^ γ-PGA; and T3—200 mg·L^−1^ γ-PGA. All fruits were then stored at 4 °C for 14 days. Different lowercased letters represent significant differences among different treatments at *p* < 0.05.

All treatments with γ-PGA showed significant effects on the activities of SOD, POD, and CAT in fruit under cold storage after 8 and 14 days of treatment, compared with the control (Figure 3). Among the different concentrations of γ-PGA, 100 mg·L^−1^ γ-PGA showed better effects on the activities of the above enzymes than other γ-PGA concentrations. After 8 days of treatment, 100 mg·L^−1^ γ-PGA increased the activities of SOD, POD, and CAT by 88.2%, 28.5%, and 93.8%, compared with the control. After 14 days of treatment, 100 mg·L^−1^ γ-PGA increased the activities of SOD, POD, and CAT by 58.5%, 39.3%, and 97.1%, respectively. The above results indicated that γ-PGA improved fruit antioxidant capacity by enhancing the activities of SOD, POD, and CAT under cold storage.

### 3.3. Effects of γ-PGA on the Activities of Enzymes in AsA-GSH Cycle in Fruit under Cold Storage

**Figure 4:**
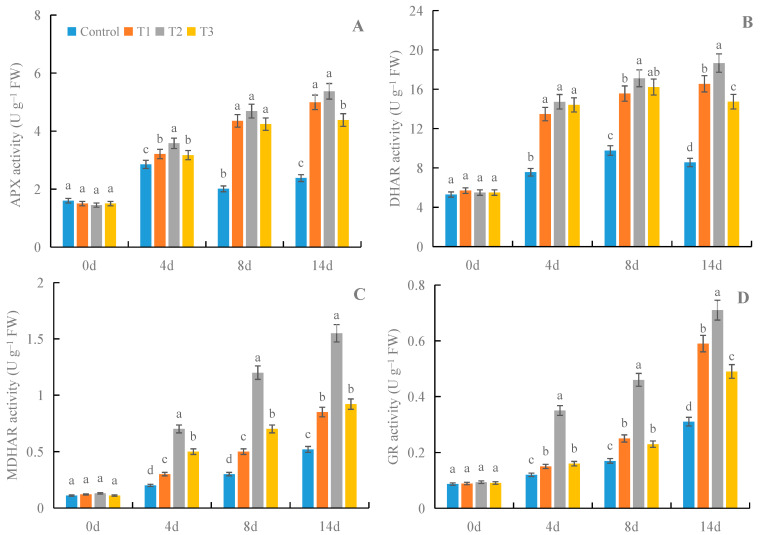
Effects of γ-PGA on the activities of APX (**A**), DHAR (**B**), MDHAR (**C**), and GR (**D**) in AsA-GSH cycle in strawberries under cold storage. The fruits were treated as follows: control—distilled water; T1—50 mg·L^−1^ γ-PGA; T2—100 mg·L^−1^ γ-PGA; and T3—200 mg·L^−1^ γ-PGA. All fruits were then stored at 4 °C for 14 days. Different lowercased letters represent significant differences among different treatments at *p* < 0.05.

Compared with the control, all treatments with γ-PGA showed significant effects on the activities of enzymes in the AsA-GSH cycle in fruit under cold storage after 4, 8, and 14 days of treatment (Figure 4). Compared with other concentrations of γ-PGA, 100 mg·L^−1^ γ-PGA showed better effects on the activities of enzymes in fruit under cold storage. After 8 days of treatment, 100 mg·L^−1^ γ-PGA increased the activities of APX, DHAR, MDHAR, and GR by 133.3%, 75.1%, 300.0%, and 170.6%, respectively, compared with the control. After 14 days of treatment, 100 mg·L^−1^ γ-PGA increased the activities of APX, DHAR, MDHAR, and GR by 125.6%, 117.9%, 198.1%, and 129.0%, respectively. The above results indicated that γ-PGA improved fruit antioxidant capacity by enhancing the activity of the AsA-GSH cycle under cold storage.

### 3.4. Effects of γ-PGA on Total Antioxidant Capacity and the Contents of MDA and H_2_O_2_ in Fruit under Cold Storage

**Figure 5:**
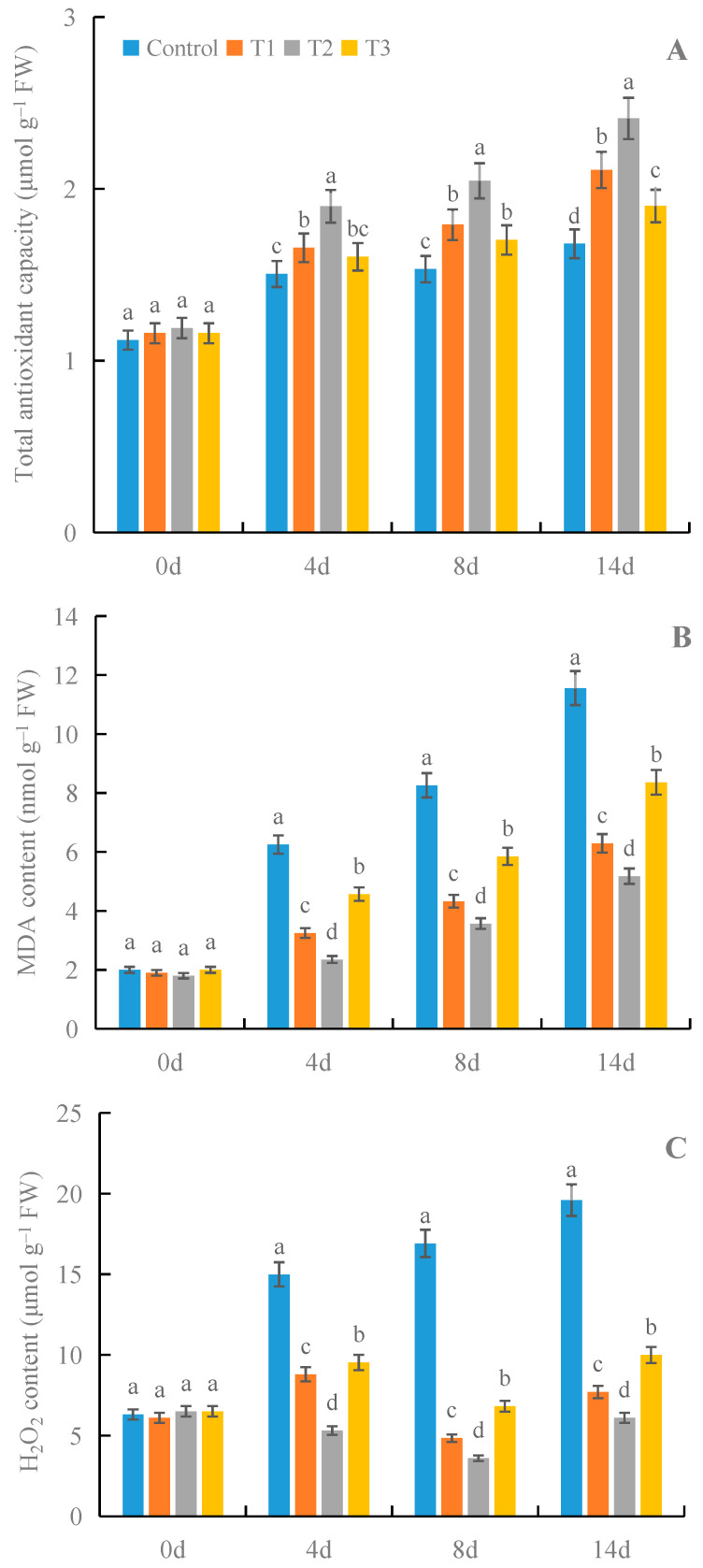
Effects of γ-PGA on total antioxidant capacity (**A**) and the contents of MDA (**B**) and H_2_O_2_ (**C**) in fruit under cold storage. The fruits were treated as follows: control—distilled water; T1—50 mg·L^−1^ γ-PGA; T2—100 mg·L^−1^ γ-PGA; and T3—200 mg·L^−1^ γ-PGA. All fruits were then stored at 4 °C for 14 days. Different lowercased letters represent significant differences among different treatments at *p* < 0.05.

All treatments with γ-PGA significantly improved the total antioxidant capacity and decreased the contents of MDA and H_2_O_2_ in fruit under cold storage, compared with the control (Figure 5). Among the different γ-PGA concentrations, 100 mg·L^−1^ γ-PGA showed better effects on the total antioxidant capacity and the contents of MDA and H_2_O_2_ than other γ-PGA concentrations under cold storage. After 8 days of treatment, 100 mg·L^−1^ γ-PGA increased the total antioxidant capacity by 33.5%, and decreased the contents of MDA and H_2_O_2_ by 56.8% and 78.7%, respectively, compared with the control. After 14 days of treatment, 100 mg·L^−1^ γ-PGA increased the total antioxidant capacity by 43.5% and decreased the contents of MDA and H_2_O_2_ by 55.2% and 68.9%, respectively. The above results once again indicated that γ-PGA improved the antioxidant capacity of strawberries under cold storage.

### 3.5. Effects of γ-PGA on Fruit Firmness and the Activities of Enzymes Responsible for Cell Wall Degradation under Cold Storage

**Figure 6:**
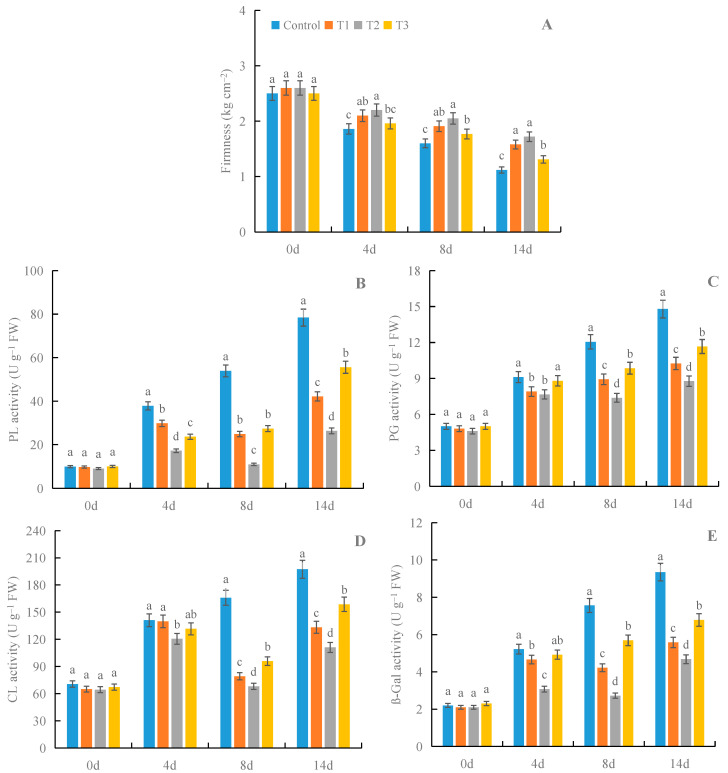
Effects of γ-PGA on fruit firmness (**A**) and the activities of PL (**B**), PG (**C**), CL (**D**), and β-Gal (**E**) in fruits under cold storage. The fruits were treated as follows: control—distilled water; T1—50 mg·L^−1^ γ-PGA; T2—100 mg·L^−1^ γ-PGA; and T3—200 mg·L^−1^ γ-PGA. All fruits were then stored at 4 °C for 14 days. Different lowercased letters represent significant differences among different treatments at *p* < 0.05.

All treatments with γ-PGA showed higher fruit firmness and lower activities of enzymes responsible for cell wall degradation than the control under cold storage, especially for 100 mg·L^−1^ γ-PGA (Figure 6). After 8 days of treatment, 100 mg·L^−1^ γ-PGA increased the firmness by 28.1%, compared with the control, and it decreased the activities of PG, PL, CL, and β-Gal by 38.7%, 79.6%, 59.0%, and 64.0%, respectively. After 14 days of treatment, 100 mg·L^−1^ γ-PGA increased firmness by 53.6% and decreased the activities of PG, PL, CL, and β-Gal by 40.7%, 66.4%, 43.7%, and 49.9%, respectively. The above results suggested that γ-PGA delayed the softening process of strawberries under cold storage by inhibiting the process of cell wall degradation through PG, PL, CL, and β-Gal.

### 3.6. Effects of γ-PGA on Fruit Color Parameters of Strawberry under Cold Storage

**Figure 7:**
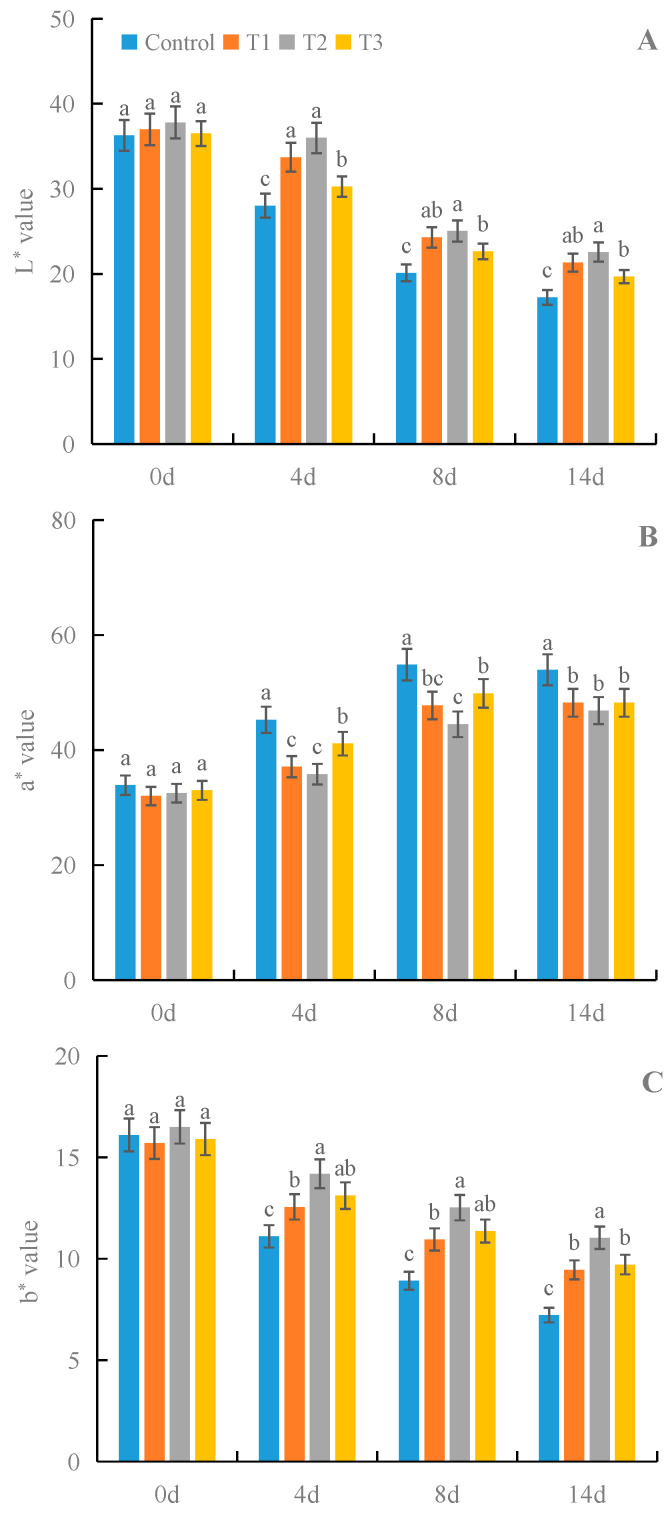
Effects of γ-PGA on fruit color parameters L* (**A**), a* (**B**), and b* (**C**) of strawberries under cold storage. The fruits were treated as follows: control—distilled water; T1—50 mg·L^−1^ γ-PGA; T2—100 mg·L^−1^ γ-PGA; and T3—200 mg·L^−1^ γ-PGA. All fruits were then stored at 4 °C for 14 days. Different lowercased letters represent significant differences among different treatments at *p* < 0.05.

All treatments with γ-PGA significantly improved the lightness value (L*) and the yellowness value (b*) of strawberries under cold storage, compared with the control (Figure 7). Among the different concentrations, 100 mg·L^−1^ γ-PGA showed better effects on the color parameters L* and b* than other γ-PGA concentrations under cold storage. After 8 days of treatment, 100 mg·L^−1^ γ-PGA increased L* and b* by 24.4% and 40.5%, respectively, compared with the control. After 14 days of treatment, 100 mg·L^−1^ γ-PGA increased L* and b* by 30.9% and 52.8%, respectively. However, all treatments with γ-PGA decreased the redness value (a*). The above results indicated that γ-PGA improved the color quality of strawberry fruits by increasing the L* and b* values under cold storage.

### 3.7. Effects of γ-PGA on Fruit Nutritional Quality of Strawberry under Cold Storage

**Figure 8:**
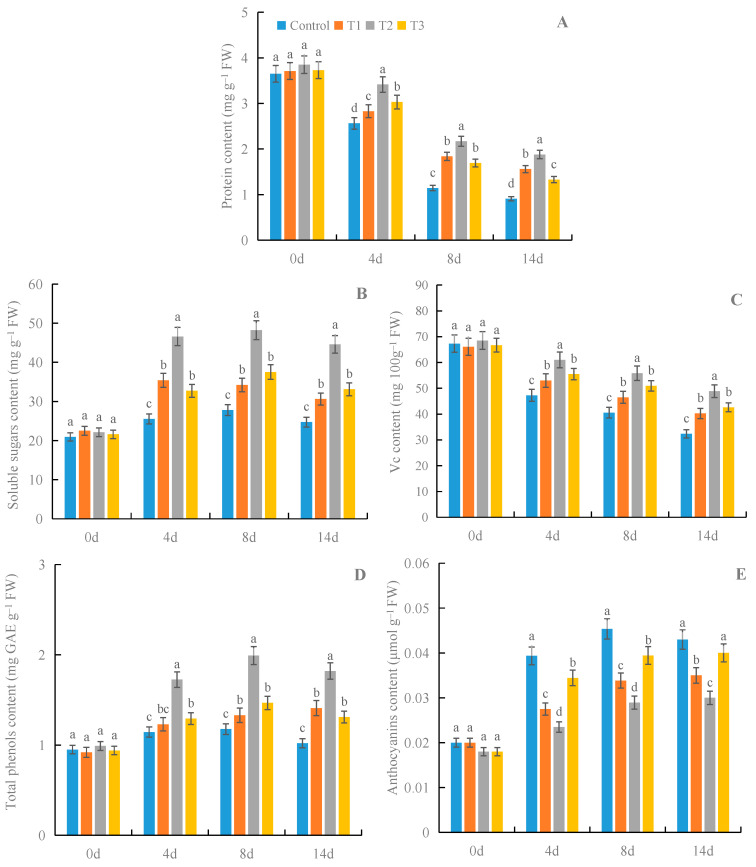
Effects of γ-PGA on the contents of protein (**A**), soluble sugars (**B**), Vc (**C**), total phenols (**D**), and anthocyanins (**E**) of strawberries under cold storage. The fruits were treated as follows: control—distilled water; T1—50 mg·L^−1^ γ-PGA; T2—100 mg·L^−1^ γ-PGA; and T3—200 mg·L^−1^ γ-PGA. All fruits were then stored at 4 °C for 14 days. Different lowercased letters represent significant differences among different treatments at *p* < 0.05.

Compared with the control, all treatments with γ-PGA significantly improved the contents of protein, soluble sugars, Vc, and total phenols, and decreased anthocyanin content in the fruits under cold storage (Figure 8). Among the different concentrations, 100 mg·L^−1^ γ-PGA showed better effects on the fruits’ nutritional quality than other γ-PGA concentrations under cold storage, except in terms of anthocyanin content. After 8 days of treatment, 100 mg·L^−1^ γ-PGA increased the contents of protein, soluble sugars, Vc, and total phenols by 89.6%, 73.1%, 37.6%, and 69.2%, respectively, compared with the control. After 14 days of treatment, 100 mg·L^−1^ γ-PGA increased the contents of protein, soluble sugars, Vc, and total phenols by 106.6%, 80.6%, 51.2%, and 78.4%, respectively. The above results suggested that, except for anthocyanin content, γ-PGA improved fruit nutritional quality under cold storage.

### 3.8. Effects of γ-PGA on the Contents of SSs and TAs and the SAR of Strawberry Fruit under Cold Storage

**Figure 9:**
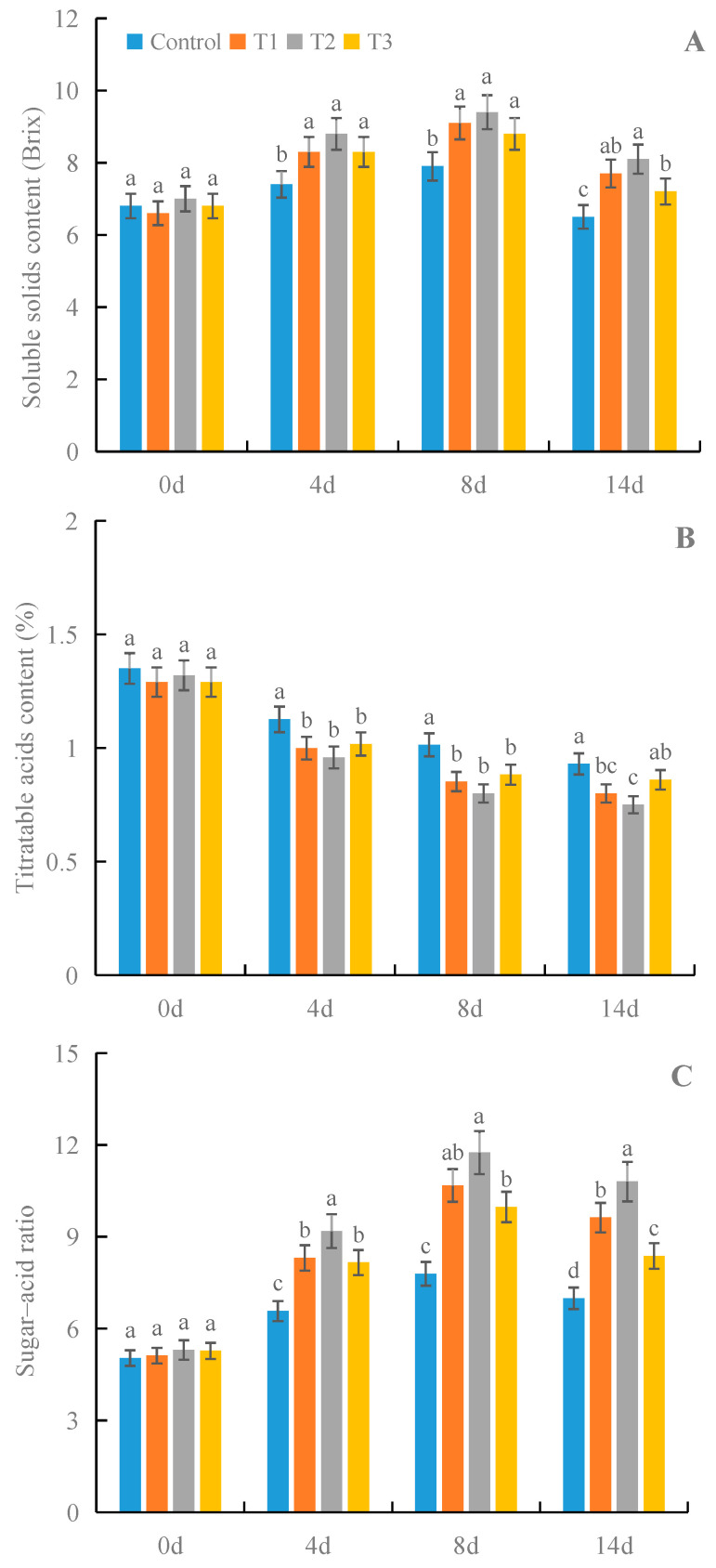
Effects of γ-PGA on the contents of SS (**A**) and TA (**B**) and the SAR (**C**) of strawberries under cold storage. The fruits were treated as follows: control—distilled water; T1—50 mg·L^−1^ γ-PGA; T2—100 mg·L^−1^ γ-PGA; and T3—200 mg·L^−1^ γ-PGA. All fruits were then stored at 4 °C for 14 days. Different lowercased letters represent significant differences among different treatments at *p* < 0.05.

All treatments with γ-PGA showed significant effects on the contents of SSs and TAs and the SAR of fruits under cold storage, compared with the control (Figure 9). Among the different concentrations, 100 mg·L^−1^ γ-PGA showed better effects on these taste indicators than other γ-PGA concentrations. After 8 days of treatment, 100 mg·L^−1^ γ-PGA increased the SS content by 19.0% and decreased the TA content by 21.1%. In addition, 100 mg·L^−1^ γ-PGA increased the SAR by 50.9%. After 14 days of treatment, 100 mg·L^−1^ γ-PGA increased the SS content by 24.6% and decreased the TA content by 19.4%, and it increased the SAR by 54.3%. The above results indicated that γ-PGA improved the SAR of strawberries by increasing the SS content and decreasing the TA content under cold storage.

## 4. Discussion

Decay and weight loss are usually used to evaluate the freshness of strawberries during cold storage. It has been documented that exogenous chemicals can reduce the decay and weight loss of strawberries. Martínez et al. [18] reported that edible chitosan coatings incorporated with *Thymus capitatus* essential oil significantly reduced the decay and weight loss of strawberries during cold storage. Bahmani et al. [4] found that proline-coated chitosan nanoparticles showed better effects on the decay control and weight loss of strawberries. For γ-PGA, Tao et al. [15] showed that γ-PGA hydrogel could improve the decay control and lower weight loss of shiitake mushrooms. However, there is still no report on the effects of γ-PGA on the decay and weight loss of strawberries during cold storage. The findings of the current study demonstrated that γ-PGA also significantly decreased the decay and weight loss of strawberries, which indicates that it is feasible to apply γ-PGA in preserving the quality and reducing the postharvest loss of strawberries during cold storage.

Many exogenous chemicals can improve strawberry preservation by enhancing its antioxidant capacity [4,7,9]. This study found that γ-PGA enhanced the activities of SOD, POD, CAT, APX, DHAR, MDHAR, and GR, which further decreased the contents of MDA and H_2_O_2_. In this way, γ-PGA enhanced the antioxidant capacity of strawberries under cold storage, which helped to preserve the strawberries for a longer period. Previous studies showed that γ-PGA enhanced the antioxidant capacity of crop seedlings under stress by increasing the activities of SOD, POD, CAT, and APX [19,20,37]. In the current study, we also found that γ-PGA enhanced the antioxidant capacity of strawberries through SOD, POD, CAT, and APX, which is consistent with previous studies on crop seedlings [19,20,37]. In addition, we also showed that γ-PGA increased the activities of DHAR, MDHAR, and GR in strawberries during storage, which suggests that γ-PGA can also improve the antioxidant capacity of strawberries by activating the AsA-GSH cycle. The above results report for the first time the roles of γ-PGA in improving the quality of strawberries by enhancing their antioxidant capacity during cold storage. It has been documented that phytohormones brassinolide (BR) and jasmonic acid (JA) and signaling molecules Ca^2+^ and H_2_O_2_ could enhance plant antioxidant capacity by regulating antioxidant enzymes [38,39,40]. Previous reports also showed that there were crosstalks between BR, JA, Ca^2+,^ and H_2_O_2_ in the regulation of antioxidant capacity in *Brassica napus* L. seedlings with γ-PGA under salt and cold stress [41]. Xu et al. [20] found that γ-PGA enhanced drought tolerance by promoting abscisic acid (ABA) accumulation in plants. However, it is still unclear whether there are crosstalks between ABA, BR, JA, Ca^2+^, and H_2_O_2_ in the regulation of antioxidant capacity in strawberry fruit by γ-PGA during cold storage. Therefore, it will be very interesting to carry out this aspect of the research to elucidate additional mechanisms for the role of γ-PGA in preserving strawberry fruit during cold storage.

Firmness is an important indicator to evaluate the quality of strawberries during storage. This study showed that γ-PGA inhibited the firmness loss of the fruits during cold storage, which indicated that γ-PGA plays a significant role in maintaining firmness. It has been documented that fruit firmness is closely related to the softening process. In strawberries, excessive softening increases their susceptibility to decay and deterioration. Thus, the finding of the current study indicated that γ-PGA delayed the softening process of strawberries. Previous studies demonstrated that the fruit softening process was closely related to the activities of enzymes responsible for cell wall degradation. Wang et al. [3] found that chitosan coating inhibited the softening of strawberry fruit during cold storage by decreasing the activities of enzymes responsible for cell wall degradation. The findings of the current study showed that γ-PGA could decrease the activities of enzymes responsible for cell wall degradation, including PG, PL, CL, and β-Gal. Therefore, our results suggest that γ-PGA can delay the softening process by decreasing the activities of enzymes responsible for cell wall degradation, which further inhibits fruit firmness loss during cold storage. The above results report for the first time the roles of γ-PGA in preserving strawberry fruit by inhibiting the firmness loss and delaying the softening process during cold storage. It was found that ABA could accelerate the softening process by enhancing the transcript levels of cell wall disassembly genes, which further decreased the fruit firmness of *Fragaria chiloensis* [42]. Xu et al. [20] demonstrated that γ-PGA promoted ABA accumulation in plants. However, we found that γ-PGA inhibited the firmness loss and delayed the softening process during cold storage. Thus, our results indicated that γ-PGA may retard fruit softening by inhibiting the signal transduction of ABA in regulating the softening process of strawberry fruit during cold storage. However, it is still unclear whether this is the case. In addition, previous studies showed that signaling molecules Ca^2+^ and H_2_O_2_ and phytohormones BR and JA participated in the regulation of antioxidant capacity by γ-PGA in *Brassica napus* L. seedlings under stress through crosstalk [41]. However, it is still unclear whether there are also crosstalks between BR, JA, Ca^2+^, and H_2_O_2_ in the regulation of the fruit softening process of strawberries by γ-PGA during cold storage. Thus, it will also be very interesting to carry out this aspect of the study to elucidate additional mechanisms that highlight the roles of γ-PGA in preserving strawberries during cold storage.

Surface color is an important quality parameter for strawberries. The lightness value (L*) is affected by the drying and browning of fruit [7,22]. We found that γ-PGA significantly increased the L* value, which indicated that γ-PGA delayed the drying and browning of the fruit surface during cold storage. This phenomenon can be proven by the reduction in weight loss and the decay rate of strawberry fruit in this study. The red color is closely related to the accumulation of anthocyanins in fruits. In addition, the accumulation of anthocyanins is closely associated with the ripeness and senescence of strawberries. We found that γ-PGA significantly decreased the value of redness (a*), which suggests that γ-PGA delays the senescence of ripe fruits during cold storage. This phenomenon can be proven by the reduction in the accumulation of anthocyanins and the increase in the shelf life of strawberry fruits in this study. Lu et al. [43] reported that the value of yellowness (b*) was affected by changes in the contents of chlorophyll, carotenoids, and flavonoids. We found that γ-PGA significantly improved the b* value, which indicates that γ-PGA decreased the content of chlorophylls and improved the contents of carotenoids and flavonoids in the fruits during cold storage. As important antioxidants, the increases in the contents of carotenoids and flavonoids can delay fruit senescence. Thus, our above findings report for the first time the roles of γ-PGA in improving strawberry preservation by decreasing the accumulation of anthocyanins and delaying fruit senescence during cold storage.

The maintenance of the nutritional quality is also important for the freshness of strawberry fruit during cold storage. Increasing lines of evidence demonstrate that many exogenous chemicals can improve the preservation of strawberries by maintaining their nutritional quality [4,5,21]. The results of the current study showed that γ-PGA maintained nutritional quality by improving the contents of protein, soluble sugars, Vc, and total phenols. However, γ-PGA decreased the content of anthocyanins. The accumulation of anthocyanins is closely related to the ripeness and senescence of strawberries. Thus, γ-PGA could delay the senescence of strawberries by reducing the content of anthocyanins, which further improved fruit preservation under cold storage. Therefore, not only can γ-PGA maintain the nutritional quality of strawberries, but it can also improve their shelf life. Our results report for the first time the roles of γ-PGA in improving the shelf life of strawberries by maintaining their nutritional quality during cold storage. The contents of nutritional quality indicators are determined by their respective metabolic enzymes. Thus, it will be interesting to investigate the roles of γ-PGA in regulating metabolic enzymes responsible for the biosynthesis and degradation of the previously mentioned nutritional quality indicators, which will shed light on additional mechanisms for improving strawberry preservation during cold storage with the use of γ-PGA.

The fruit taste is an important sensory quality. The sugar–acid ratio is an important indicator for evaluating the fruit taste of strawberries. This ratio is determined by the contents of SSs and TAs. Previous studies demonstrated that exogenous chemicals could improve the SAR by increasing the SS content and decreasing the TA content in fruit [44]. Our results also showed that γ-PGA improved the SAR by increasing the SS content and decreasing the TA content in fruit during cold storage, which is in agreement with the results of a previous study [44]. The current study revealed that γ-PGA could improve sensory quality by increasing the SAR. Previous studies showed that the contents of SSs and TAs are determined by their respective metabolic enzymes. Thus, it will be interesting to investigate the roles of γ-PGA in regulating the metabolic enzymes responsible for the biosynthesis and degradation of SSs and TAs, which can provide further insights for improving strawberry preservation during cold storage with the use of γ-PGA.

As a non-toxic, edible, biodegradable, and eco-friendly biopolymer, γ-PGA has been used to prepare γ-PGA hydrogel by chemical crosslinking with polyethylene glycol diglycidyl ether in the food industry [15]. Tao et al. [15] reported that γ-PGA hydrogel could improve the storage life and quality of shiitake mushrooms by decreasing weight loss, decay rate, and Vc loss. However, there are still no reports on the application of γ-PGA in the preservation of strawberries during storage alone or in combination with other chemicals. Therefore, the findings of the current study provide significant insights into the application of γ-PGA in the preservation of strawberries during storage. In addition, we found that 200 mg·L^−1^ γ-PGA was not as effective as 100 mg·L^−1^ γ-PGA in most of the evaluated traits for strawberry preservation. This phenomenon indicates that the effect of γ-PGA on the preservation of strawberries is not positively correlated with its concentration. In this study, we found that 50 and 100 mg·L^−1^ γ-PGA had better effects on fruit preservation than 200 mg·L^−1^ γ-PGA, in particular 100 mg·L^−1^ γ-PGA. Thus, a suitable concentration of γ-PGA should be selected through experiments in its application for strawberry preservation. In this study, our results suggested that 100 mg·L^−1^ γ-PGA was the suitable concentration of γ-PGA in improving the preservation of strawberry cv. Hongyan under cold storage.

## 5. Conclusions

In conclusion, our results clearly showed that γ-PGA could effectively improve decay control and maintain the quality of strawberry fruit under cold storage. γ-PGA improved decay control by enhancing the antioxidant capacity through SOD, POD, CAT, APX, DHAR, MDHAR, and GR. γ-PGA reduced the firmness loss and delayed the softening process by decreasing the activities of PG, PL, CL, and β-Gal. γ-PGA improved the color quality by increasing the values of L* and b*. γ-PGA maintained the nutritional quality by increasing protein, soluble sugars, Vc, and total phenol contents. γ-PGA improved the SAR by increasing the SS content and decreasing the TA content in fruits. These results suggest that γ-PGA significantly contributes to improving the quality of strawberry cv. Hongyan under cold storage.

## Data Availability

The data used to support the findings of this study can be made available by the corresponding author upon reasonable request.
